# Influence of Salting Technology on the Diffusion of NaCl in Swordfish (*Xiphias gladius*) Fillets

**DOI:** 10.3390/foods11020164

**Published:** 2022-01-09

**Authors:** Francesco Corrias, Efisio Scano, Giorgia Sarais, Alberto Angioni

**Affiliations:** 1 Food Toxicology Unit, Department of Life and Environmental Science, Campus of Monserrato, University of Cagliari, 09042 Cagliari, Italy; Francesco.corrias@unica.it (F.C.); gsarais@unica.it (G.S.); 2 Faculty of Agraria, University of Sassari, 07100 Sassari, Italy; efisiscano@gmail.com

**Keywords:** salt, food processing, swordfish, dry salting, injection salting

## Abstract

Swordfish is the most widespread billfish in the aquatic environment. The industrial processing of swordfish fillets involves salting, drying, and smoking steps. Salting techniques, dry or wet, are the most common method of fish preservation. This work evaluated salt diffusion in swordfish fillets after traditional dry salting and wet industrial injection salting methods. The data obtained from the dry salting studies highlighted that the salt diffusion process in swordfish meat was an unfavorable process depending on the contact time with the salt/meat. Moreover, irregularly shaped fillets negatively affected the salt migration in the different areas, leading to inhomogeneous and possibly unsafe final products. On the contrary, wet injection salting was suitable for processing swordfish fillets. As a result, the final products had a homogeneous salt concentration, maintained the organoleptic characteristics and health benefits for a long period, and achieved a longer shelf-life. Furthermore, the water activity (aw) values detected for the different processed fillets confirmed the physicochemical features of the final products and allow the classification of safe products. Moreover, injection salting is a quick process compatible with industrial production times.

## 1. Introduction

Swordfish (*Xiphias gladius*), a pelagic fish of the *Xiphidae* family, live in different marine environments, such as the Atlantic, Pacific, and Indian oceans and the temperate waters of the Mediterranean Sea [1]. Different processing technologies can be applied to swordfish, such as salting, drying, smoking, and marinating, to extend market shelf life [2]. Salting was the first technology reported by Egyptians and Chinese [3]; it extends food storage time, avoids possible contamination by pathogens, and confers a stronger taste and flavor [4]. Salting acts by removing the water from cells, which decreases the water activity (aw) of the medium, creating an environment not suitable for microorganism development and leading to their death [5]. However, excessive intake of high-salt foods has been associated with hypertension, cardiovascular diseases, and nasopharyngeal carcinoma [6,7]; therefore, strategies intended to decrease salt in food by optimizing its diffusion technologies are needed. Dry salting, injection of salt solutions, and brining are the most used [8]. The diffusion of salt is driven by the NaCl gradient between the outer and inner layers of the muscle. It depends on fish species, fat content, muscle fiber, skin, fillet thickness, temperature, salting technology, state and quality of the meat, and the surface:volume ratio of the meat [9]. Brining is commonly used to preserve fermented vegetable products, such as sauerkraut and gherkins, and to prepare table olives and cheese. Typical brines have NaCl concentrations of 5–10% (weak), 18% (medium), and 25–30% (strong). The direct injection of salt solutions to the muscle through industrial multi-needle equipment can give more efficient salt distribution [10]. Dry and wet salting effects have been evaluated in different fish species, such as sardine, Atlantic cod, salmon, European sea bass, and bighead carp [3,8,9,10,11,12,13,14,15]. No studies were found in the literature that address salt diffusion in swordfish fillets after dry salting and wet salting by direct injection of salt solution into the muscles. This work aimed to evaluate the distribution of salt in swordfish fillets after dry salting and wet salting by manual and industrial injection. Five trials were performed to examine different salting methods, storage time and temperature, and final processing.

## 2. Materials and Methods

### 2.1. Standard and Reagents

A medium grain of sea salt was purchased from the market and used for dry salting and preparing brines. HNO_3_ 65%, AgNO_3_ 0.1 N (NORMEX), ferric alum, and NH_4_SCN 0.1 N (NORMEX) were purchased from Carlo Erba (Milan, Italy). Brines were prepared at 32.5% (*w*/*v*) and 7% (*w*/*w*) for manual and industrial injection trials by solubilizing NaCl in Milli-Q water. The double-distilled water was obtained using a Milli-Q water purification system (Millipore, Bedford, MA, USA).

### 2.2. Swordfish Sample Processing

Fresh swordfish fillets, homogeneous in size and shape, were supplied by a fish processing company. Five conditions were examined: (a) industrial dry salting and drying; (b) dry salting and storage at 7 °C; (c) dry salting, rinsing, and storage at 7 °C; (d) dry salting vs. manual wet salting and storage at 18 °C and 7 °C, vs. dry salting plus manual wet salting and storage at 7 °C; (e) industrial wet salting, and industrial wet salting plus drying and smoking.

Dry salting was performed by sprinkling with medium-sized salt and manual massage. Wet salting (2 mL of 32.5% NaCl) was performed manually using a medical polypropylene syringe with a 21G steel needle (inserted 4 cm in the muscle); the solution was administered at nine points at a distance of 4 cm longitudinally and 2.5 cm transversally. Industrial wet salting was performed using a multi-needle apparatus (Ruhle–PR 20, Grafenhausen, Germany) with 2 × 10 vertical steel needles (diameter 2 mm, length 160 mm) with an internal space of 0.8 cm. The plunger allowed the simultaneous penetration of the needles inside the swordfish samples, transported by a sliding roller. Injection pressure and volume and needle-block velocity were 1.5 bar, 0.6 L, and 30 strokes per min, respectively. The brine (7% in NaCl) was introduced into the muscles at room temperature. After injection, fillets were placed on a grid for 1 min to drain the excess brine and stored at 4 °C. The industrial drying process consists of 8 days at 17 °C, and the industrial smoking process was 4 h at 24 °C + ½ h at 50 °C.

Fillets had an average weight of 3565 ± 8.81 (g ± RSD%), a total length of 40 cm, a width of 16 cm, and a height of 8.7 cm. Each fillet was divided into three equal parts before processing in studies a, b, and d. In contrast, in study c, fillets were divided in two groups. Portion of fillets of 420 g were processed unmodified in the first group, whereas in the second were cut to produce cubic-shaped portions with the following dimensions: total length 13 cm, width 7.5 cm and height 5.2 cm. In study e, each fillet was divided into eight slices that were analyzed separately.

(a) Industrial dry salting, rinsing and drying. Twenty swordfish fillets were subjected to dry salting according to the procedure used by the company. The fillets thawed at room temperature (18 °C) were left to drain and salted by massage. The excess of salt on the surface was eliminated by rinsing before drying.
(b) Dry salting + storage at 7 °C. Thirty swordfish portions were subjected to salting by massage and stored in a cell at 7 °C. Samples were collected after 1, 2, 5, and 8 h. At each sampling time, the excess salt was removed by brushing.
(c) Dry salting, rinsing and storage at 7 °C. Thirty swordfish portions were subjected to dry salting and stored in a cell at a controlled temperature (7 °C). Samples were collected at 1, 2, and 6 h, rinsed with Milli-Q water, wiped, and stored in a refrigerated cell for 24 h.
(d) Dry salting vs. manual wet salting and storage at 18 °C and 7 °C vs. dry salting + manual wet salting and storage at 7 °C. Twenty swordfish were cleaned and subjected to (1) dry salting; (2) manual wet salting; (3) combination of (1) and (2) salting processes. All the samples were then stored at a controlled temperature of 7 °C in a cold store for one week. A representative fraction was taken from each slice, carefully homogenized, and analyzed to determine the amount of NaCl.
(e) Industrial wet salting + industrial drying and smoking. Twenty swordfish fillets were subjected to industrial injection. After that, fillets were separated into three groups, (1) was used for NaCl analysis after wet salting; (2) was subjected to drying; (3) was subjected to drying and smoking.

After processing, the fillets were finely ground, homogenized, and compared with untreated fillets (study a). In addition, in studies b, c, and d, fillets were dissected in subsamples separating the lateral, frontal, superior, and inferior slices (external portion) from the inner district (internal part) and the central portion (heart). Sub-samples were finely chopped and homogenized individually before analysis.

Each processed fillet represented a single replicate.

### 2.3. Salt Content and Water Activity

NaCl content was determined according to the Volhard method. One gram of swordfish pulp was weighed, placed in a 250 mL conical flask with 20 mL of a standard solution of AgNO_3_ 0.1 N + 20 mL HNO_3_ (65% in water), and hot-digested under constant stirring. Digestion was considered finished when no traces of the sample were found and a white precipitate of AgCl settled on the bottom of the flask. Fifty milliliters of Milli-Q water and 2 mL of ferric alum were added to the solution as an indicator. The excess silver ions (Ag+) were titrated using a standard solution of 0.1 N ammonium thiocyanate. The amount of salt was expressed as %/fresh weight (FW).

Water activity (aw) was determined using an Aqualab 4-TE water activity meter (Meter Group, Milan, Italy).

### 2.4. Statistical Analysis

Analysis of variance (ANOVA) was carried out with the software XLSTAT (Addinsolf L.T.D., v. 19.4); mean comparisons of the effects of treatments were calculated by the Fisher’s least significant difference test at *p* ≤ 0.05.

## 3. Results

The fresh swordfish fillets analyzed in the study (a) had a NaCl content of 0.26 ± 9.31 (% ± RSD%), whereas the average value after the industrial process was 2.39 ± 5.29 (% ± RSD%) (Table 1). The aw was 0.99 and 0.95, before and after processing, respectively.

In study (b), the salt content of the fresh control fillets was 0.17 ± 4.69 (% ± RSD%). Dry salting led to a concentration of NaCl in the external layer ranging from 2.86 ± 33.5 (% ± RSD%) to 5.80 ± 8.3 after an eight-hour experiment (Table 2). On the contrary, the NaCl concentration in the internal part was about five times lower, ranging from 0.56 ± 29.6 and 1.34 ± 3.4 (% ± RSD%). It was homogeneous in the first five hours, but increased in the last storage step (Table 2). Finally, the heart of the fillet had NaCl levels overlapping the control samples. After eight hours, a small NaCl increase was registered, but far below the surrounding parts of the muscle (Table 2).

Two groups of fillets (study c) were selected to evaluate the influence of the shape on NaCl distribution. The first group was processed as it was; the second group was cut to obtain regular cubes. The samples of the first group had an average NaCl concentration in the external portions ranging from 1.74 ± 27.15% to 3.51 ± 27.95% (Table 3). These values were slightly lower than those found in study (b), which was due to the rinsing step with water. The values for the internal layer and the heart were in line with the previous study (Table 2 and Table 3). Swordfish samples uniform in shape and size (second group) had average NaCl concentrations higher at each sampling time and in each district, showing better salt diffusion in the muscle (Table 3). 

In the study (d), the samples were stored after the salting process for one week at 7 °C and 18 °C. After dry salting, the NaCl concentration showed a gradient from the external layer to the heart of the fillets (Table 4). However, an evident difference was observed only between the external layer and the heart of the muscle. On the other hand, manual injection fillets of at 7 °C showed the inverse situation. The heart of the fillet had the highest salt concentration (2.44 ± 12.4%) with the concentration decreased towards the external layer (Table 4). Wet salting at 18 °C resulted in the same trend; however, NaCl levels were more than a factor of two lower (Table 4). When combining the two processes, the obtained subsamples showed overlapping NaCl values, with values comparable to the sum of the single salting processes (Table 4).

The NaCl content in study (e) showed a homogeneous distribution in the selected slices (Table 5). When comparing the amount of NaCl after wet salting vs. dried and smoked fillets, an increase in salt concentration for an average of 25 and 34% was observed, which agreed with the weight loss in the fillets (Table 5). The water activity (aw) value of the smoked fillets was 0.90 ± 3.24. The fillets that were not entirely defrosted (ND) were less efficient in retaining the brine, leading to lower NaCl values (Table 5).

## 4. Discussion

Salting is the most common preservation technique used to increase the shelf-life of fish and fish products, with cod, salmon, and sardine the most processed [8,12,13,14,16]. Many papers reported the effect of salting on product yields and characteristics after cooking [13] and the fat content [14]. Moreover, Birkeland et al. [16] investigated the instrumental parameters (needle speed, injection pressure, number of repeated injections, fillet resting time before smoking, post-salting, and post-smoking yields) of wet industrial salting by injection into Atlantic salmon fillets. Wet salting trials with NaCl brine at 25% resulted in final values of about 4% [15]. On the other hand, studies on salmon found NaCl values of 1% after smoking, similar to those obtained in our study [10]. However, no previous studies were performed on swordfish fillets.

Excess dietary salt is considered the most critical factor responsible for essential hypertension [17]. The World Health Organization (OMS) recommends levels of sodium ≤2 g/d, corresponding to about 5 g of table salt [18]. Considering an average daily consumption rate of salted pelagic fish of about 12 g/day [19], the levels obtained after dry salting (study c) and wet industrial processing (study e) allowed consumption of processed swordfish of almost 250 g/d. However, the total salt intake from other food commodities should be evaluated to correct the serving size.

This paper examines NaCl diffusion in swordfish fillets following different salting approaches to reduce the NaCl content in food and improve human health.

The obtained data show that the contact time affects the salt diffusion into the muscle during the dry salting process. However, the distribution was also dependent on temperature and thickness, according to the literature [20].

The salt naturally spreads throughout the muscle following a concentration gradient among the outer and inner layers, resulting in a time-consuming and unfavorable process [21]. The long time required for dry salting is not suitable for industrial procedures; moreover, this could lead to a final product with non-homogeneous distribution, resulting in possible harmful effects on human health [10]. In addition, the NaCl concentration found in the heart of the fillets had similar values to those of the untreated fillets and high aw levels suitable for microbial development. The poor homogeneity of the migration of salt inside the muscle is considerably affected by irregularly shaped fillets, with the tighter parts containing higher concentrations of salt.

Furthermore, when the excess salt was removed by rinsing, the muscle softened excessively, creating an ineffective predisposition to drying. The solubilization of the salt in the water during the rinsing phase led to the significant removal of NaCl. Better results were obtained by gentle brushing.

Good commercially smoked fish products require minimizing the inhomogeneity of salt content throughout the fish meat [10]. Direct injection of brine into the muscle can lead to a homogeneous distribution, reducing the diffusion time of the salt in the muscle.

**Figure 1:**
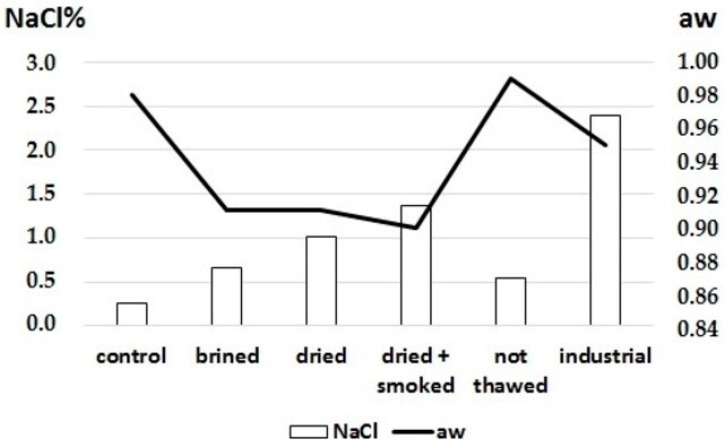
NaCl% content and aw values after different treatments were applied to swordfish fillets. aw: water activity.

However, the process is strictly related to the brine concentration and the muscle characteristics. The water-holding capacity is linked to muscle structure and freshness [22]; moreover, fillets with high fat content or not correctly defrosted have a lower propensity to retain the brine, preventing its diffusion in the muscle and losing a significant part of the injected solutions [23]. An incorrect freezing process can generate large water crystals inside the muscle, causing the cell membranes to break and the intracellular moisture to leak. In addition, a decrease in the water-holding capacity (WHC) of the muscle during the thawing step was observed, influencing its ability to retain fluid [24]. After the injection process, the incompletely thawed muscle could not maintain the brine, leading to lower NaCl values (Figure 1).

Moreover, the diameter of the needles and the brine flow affected the final results. Needles should be sized to avoid damage to the muscle structure, and the flow should be adjusted to avoid holes in the fibers. The use of needles with a diameter greater than 2 mm caused the development of visible lesions on the fresh fillets, affecting product quality.

Wet salting performed by industrial injection of 7% brine led to homogeneous NaCl values throughout the different sections of the fillets. Moreover, after the drying and drying/smoking process, NaCl levels increased proportionally with the water loss (Figure 1). These data are in accordance with the previous results obtained from salmon fillets [10].

The analyses carried out on the fillets subjected to the standard industrial process revealed higher NaCl values (Figure 1); however, water activity (aw) values remained high (0.95), meaning that a reliable shelf-life could not be obtained from a microbiological point of view. This result was attributable to the variable NaCl distribution in the muscles with aw values below 0.90 in the external parts and above 0.98 in the inner layers. Water activity represents the water available in a portion of food that microorganisms can use for growth; thus, aw can be modulated by modifying the moisture content or increasing solute concentrations. Moreover, since microbial cells consist of high solute concentrations surrounded by semipermeable membranes, the osmotic effect produced by the food environment is essential for determining their fate [24]. Bacteria result in the most sensitive microorganisms and are inhibited at a water activity in the range of 0.90–0.91. In addition, aw values of 0.60 or lower can stop the development of any pathogen [25].

This study obtained acceptable aw levels only after wet mechanical injection salting followed by industrial drying and smoking. The average value of aw was 0.90, which, when combined with reduced levels of NaCl, resulted in a safe product for human health.

## 5. Conclusions

The data obtained from the traditional dry salting studies highlighted that the diffusion process of salting in swordfish is particularly unfavorable and closely related to the contact time with salt/meat. Therefore, a suitable contact time should not exceed half a day; moreover, after the salting process, fillets should be stored at 7 °C to avoid spoilage phenomena.

Irregularly shaped fillets can have further negative effects on salt migration in the different districts, leading to inhomogeneous final products, generating possible microbial contamination that is dangerous for consumer health, and causing off-flavors.

Moreover, wet salting by injection was suitable for the treatment of swordfish fillets. The final products had homogeneous salt concentrations, maintaining the organoleptic and health benefits for long periods when stored below 7 °C. Moreover, wet salting is a quick process compatible with the production times of industrial companies. The choice of the brine concentration is crucial to optimize the salt amounts in the final product. At the same time, the size of the needles and the pressure applied should be taken into account to avoid possible structural damage to the fillet and the loss of brine.

## Figures and Tables

**Table 1:** Concentration of NaCl after the industrial process.

	NaCl (% ± RSD%)	aw ± RSD%
Fresh	0.26 ± 9.31	0.99 ± 0.31
Processed	2.39 ± 5.29	0.95 ± 1.45

aw: water activity.

**Table 2:** NaCl concentration (%) in swordfish fillets after dry salting and storage for different times.

Portion	Collection Times							
	1 h	aw	2 h	aw	5 h	aw	8 h	aw
External	2.86 ^a^	0.90	4.15 ^b^	0.86	4.20 ^b^	0.86	5.80 ^c^	0.83
Internal	0.56 ^a^	0.96	0.65 ^a^	0.97	0.75 ^a^	0.96	1.34 ^b^	0.91
Heart	0.09 ^a^	0.99	0.13 ^a^	0.99	0.27 ^ab^	0.99	0.31 ^b^	0.99

^a–c^ In each row, means followed by a common letter are not significantly different by Fisher’s least significant difference (LSD) procedure, *p* ≤ 0.05.

**Table 3:** NaCl concentration (%) in swordfish fillets after dry salting followed by rinsing and storage.

Portion	Collection Times											
	First Group						Second Group					
	1 h	aw	2 h	aw	6 h	aw	1 h	aw	2 h	aw	6 h	aw
External	1.74 ^a^	0.91	2.24 ^a^	0.90	3.51 ^a^	0.87	2.66 ^a^	0.90	2.84 ^a^	0.90	4.64 ^b^	0.84
Internal	0.54 ^a^	0.97	0.59 ^a^	0.97	0.76 ^a^	0.96	0.96 ^b^	0.94	0.63 ^a^	0.97	0.91 ^a^	0.95
Heart	0.06 ^a^	0.99	0.07 ^a^	0.99	0.11 ^a^	0.99	0.41 ^b^	0.98	0.62 ^c^	0.97	0.84 ^d^	0.96

^a–d^ In each row for each treatment, means followed by a common letter are not significantly different by Fisher’s least significant difference (LSD) procedure, *p* ≤ 0.05.

**Table 4:** NaCl concentration (%) after dry salting, manual injection, wet salting, and dry + wet salting stored for one week at 7 °C and 18 °C.

Portion	Experiments			
	Dry (7 °C)	Wet (7 °C)	Wet (18 °C)	Dry + wet (7 °C)
External	5.08 ^a(a)^	1.53 ^b(a)^	0.66 ^c(a)^	6.62 ^d(a)^
Internal	4.47 ^a(ab)^	2.10 ^b(ab)^	0.74 ^c(a)^	6.14 ^d(a)^
Heart	3.34 ^a(b)^	2.44 ^b(b)^	1.20 ^c(b)^	5.30 ^d(b)^

^a^^–d^ Values among rows (without brackets) followed by different letters differ significantly by Fisher’s least significant difference (LSD) procedure, *p* ≤ 0.05. ^(a),(b), (ab)^ Values among columns (in brackets) followed by different letters differ significantly by Fisher’s least significant difference (LSD) procedure, *p* ≤ 0.05.

**Table 5:** NaCl concentration (%) in swordfish fillets after industrial wet salting, drying, and smoking.

Portions	ND ^z^	Wet Salted	Dried	Smoked
1	0.59 ^a(b)^	0.71 ^a(a)^	1.11 ^a(b)^	1.55 ^a(c)^
2	0.44 ^a(b)^	0.62 ^a(a)^	0.95 ^a(b)^	1.36 ^a(c)^
3	0.51 ^a(a)^	0.62 ^a(a)^	0.97 ^a(b)^	1.29 ^a(c)^
4	0.41 ^a(a)^	0.65 ^a(a)^	0.99 ^a(b)^	1.29 ^a(c)^
5	0.51 ^a(b)^	0.66 ^a(a)^	1.03 ^a(b)^	1.31 ^a(c)^
6	0.52 ^a(b)^	0.68 ^a(a)^	0.94 ^a(b)^	1.36 ^a(c)^
7	0.59 ^a(b)^	0.66 ^a(a)^	1.05 ^a(b)^	1.38 ^a(c)^
8	0.73 ^b(b)^	0.66 ^a(a)^	1.11 ^a(b)^	1.41 ^a(c)^
aw	0.99	0.91	0.91	0.90

^(a),(b),^^(c)^ Values among rows (in brackets) followed by different letters differ significantly by Fisher’s least significant difference (LSD) procedure, *p* ≤ 0.05. ^a^^,b^ Values among columns (without brackets) followed by different letters differ significantly by Fisher’s least significant difference (LSD) procedure, *p* ≤ 0.05. ^z^ Not entirely defrosted.

## Data Availability

The datasets generated for this study are available on request to the corresponding author. The study did not report any data.
